# Dr. Combe on the Observation of Nature in the Treatment of Disease

**Published:** 1847-04

**Authors:** Andrew Combe

**Affiliations:** One of the Physicians in Ordinary in Scotland to the Queen


### II. ON THE OBSERVATION OF NATURE IN THE TREATMENT OF DISEASE.

BY ANDREW COMBE, M.D.

One of the Physicians in Ordinary in Scotland to the Queen.

(*In a Letter to John Forbes, M.D., F.R.S.*)

Edinburgh, Feb. 26, 1847.

MY DEAR SIR,—Soon after the publication of your last Number, a friend put into my hands Hufeland's 'Enchiridion Medicum,' published in Berlin, in 1836, but which I had not previously seen. It is a practical work, and embodies the results of fifty years' extensive experience as a physician. The opening chapter, entitled, "Nature and Art," embodies views so much in accordance with those recently advocated in your Review, that the following translation of it may prove not uninteresting to your readers.

"'*Natura sanat, medicus curat morbos.*'

"All healing of disease is effected by Nature, art is only her assistant and cures through her.

"In the same way as an internal morbid condition of organic life—an internal process of disease—lies at the foundation of and produces the external appearances (symptoms) of every disease, even so also an internal curative process—an exertion of the organic life for changing and converting the abnormal into the normal condition—lies at the root of every curative act, and alone renders it possible.

"This holds in the case of all diseases without exception. In visible or so-called surgical diseases nobody calls it in question. Every surgeon admits that it is not he who heals a broken bone, a wound or a bruise, but that it is the vital power of Nature which especially effects the result through her wonderful processes of exudation, agglutination, suppuration, separation of the dead parts from the living, and regeneration, and that his business is only to take care that these operations be allowed to go on regularly, and all obstacles to the fulfilment of her design be removed. But precisely the same rule holds with the internal diseases, the immediate relations of which are hidden from our observation, only with this difference, that we cannot see these healing-processes and organic changes with our eyes. This is true not only as to acute diseases, viz., those attended with a considerable degree of excitement, but also as to chronic, only more slowly and in a less striking manner. In slight diseases we see daily that recovery ensues without any help from art. The same thing happens also in severe and even in the worst cases. There is no disease, from the most acute inflammatory fever to the most deadly plague—no disease of either suppression or excess in the secretions and excretions, no dynamic or other affection, which has not already been cured by Nature alone. In what way does art contribute to recovery? We bleed in inflammation, lower the tone of the system, and believe that we have thereby cured. But we have only removed the obstacles and hindrances, the superfluity of blood and of excitement, and thereby placed Nature in the condition required to complete the internal healing process which must always go on if our cure is to be effected. We support the strength of the system in adynamic and low nervous diseases, and believe thereby to make a cure; but in reality we only excite the *vis medicatrix Naturæ* sufficiently to enable her to complete the internal restorative processes, which are necessary to recovery. Even the direct cure of diseases by means of what are called specifics, is the work of Nature, as the remedy only operates antagonistically, but the reaction and change for the better thus excited, take place only through the co-operation of the internal healing power of Nature. In the *dyscrasien* also, even where a specific poison is received into the system, the healing-power of Nature may effect a recovery. Is it necessary to recall the thousands of cases in which venereal affections have been recovered from without any medicine, and even as at present with the studied avoidance of mercury? But also in the deepest seated or most severe forms of constitutional syphilis, what can mercury accomplish without the co-operation of that internal healing power, which effects the expulsion of both the poison and the remedy, and restores that normal condition of the organization which is necessary for the production of healthy secretion and nutrition? How often do we find that the use of mercury in every form is of no avail, till by the combined use of strong nourishment and tonics we have restored the energy of the vital powers to the degree required for the due operation of the internal healing-process, and even of the mercury itself!

"This internal healing-power often shows itself in those remarkable changes, crises and metastasis, which arise most unexpectedly and surprisingly from it alone, and which often at once put a stop to or modify a long and severe malady which has resisted all the efforts of art. The patient whom in the evening we believed destined to certain death, falls during the night into a profuse perspiration, and is found in the morning entirely out of danger. In a severe acute disease, against which we have in vain exhausted all our resources, an external abscess suddenly shows itself, and the disease vanishes. Yes, what crowns the *vis medicatrix Naturæ*, is her frequent victory over the most different, contradictory, and unreasonable methods of cure. Do we not see daily in the country, men recover either without assistance from art, or with only the most senseless treatment? Even under professional treatment, I have long been convinced that most cures are effected, under the advice of the physician indeed, but in a very small proportion in consequence of that advice.

"This then is the meaning of the great word *crisis*, which reaches us from hoary antiquity with so elevated and so mysterious a meaning. It is not the critical evacuations, not the consequent external change, but the internal healing-process, the internal ripening of the disease, the operation of the internal assimilating, secreting, metamorphosing and renovating power of Nature which lie at the foundation of all the external phenomena. It is this which the word expresses, and it is this which is the meaning attached to it when used by all true-to-nature, profound, and unprejudiced physicians, from Hippocrates down to Sydenham, Hofmann, and Boerhaave.

"A system of medicine which embraces Nature in this sense; which, in all that it does, recognises and respects the higher laws of life and the self-acting power of Nature; which considers itself not as the *agent* but as the *instrument* of the internal healing power; which recognises the indications for interference, and decides on acting only from the wants and claims of diseased Nature; which looks upon everything that takes place in the system, whether disease, or the results of its own curative process, or the operation of medicines, as vital actions;—in one word, which itself lives in life, and which, as it recognises every thing that lives to be elevated by life to a higher sphere of existence, confines its operation accordingly to this sphere, and so becomes one with the *vis medicatrix;*—such a system of medicine I call *physiatrik.* The meaning generally attached to this term is equivalent to the *vis medicatrix Naturæ*, but I understand it to mean *a system of medicine founded on this vis medicatrix.* This is the only true system of medicine, and is founded on the eternal laws of Nature. It is it which, from the time of Hippocrates downwards, has ever been the ideal (object) of the true *Iatricists* (?), and which, through every change of the systems of the schools, has existed in the mind of the genuine practitioner. It is it whose disciple I confess myself, and it is it to which I have always belonged.

"Hence then may be discovered the true meaning of art, its relation to nature and to the position of the physician. As certainly as the *vis medicatrix Naturæ* is the foundation of every cure, (which indeed could not be accomplished without her) so certainly is her operation lightened by art, through which alone she is sometimes rendered capable of effecting a cure. Herein lies the value and the necessity of art. The stricter conclusions from it are as follows:—

"1. Art can sometimes entirely remove the disease and render the internal action unnecessary merely by removing the exciting cause, *e. g.* by the removal of a foreign body, a poison, or any gastric accumulation which excites disease.

"2. The *vis naturæ* is occasionally so exalted, and its operation so stormy and active, that it may either exhaust itself or cause injury to some important organ. In such cases art may reduce the excitement to the degree required for the production of a perfect crisis, and for warding off dangerous accidents.

"3. Nature may on the other hand want sufficient strength to fulfil completely the internal healing process. In such cases art steps in, and by proper strengthening means, supplies and sustains the tone required, and so enables the internal sanative process to go on.

"4. Art may remove obstacles which either prevent or obstruct Nature from carrying on the internal healing process. Under this head may be arranged the important condition of a suitable diet and regimen, the quiet and repose necessary during febrile excitement; the avoiding of vitiated air, bad food, &c. &c.

"5. Art may assist Nature in her struggle against particular conditions of disease, with remedies peculiarly applicable to these conditions.

"6. Art may support Nature during a crisis which she has commenced, and enable her to complete it.

"7. Lastly: there are morbid causes and conditions unconquerable by Nature alone, *e. g.* the syphilitic poison and mechanical injuries. Here art alone is of service either by the use of remedies to counteract the effects of the poison, or by mechanical and surgical help.

"Such is the object of the art of healing, and such are its limits. The physician should not be the *magister* but the *minister Naturæ*, her servant or rather her assistant, ally, and friend Hand-in-hand he should march on with her to the fulfilment of the great work, never forgetting that it is not he but she who performs it, respecting her, having her ever before his eyes, and disturbing her as little as possible.

"There are two errors which arise from this, and against which the physician must be always on his guard.

"The first is, DOING TOO LITTLE, negative treatment, or leaving *all* to Nature. This is a fault into which the new homœopathic school in particular often falls, and which may have the most melancholy consequences where active and positive treatment is really requisite to save the patient. It is proper in itself only where no rational indication for treatment can be discovered; where only time and patience are required for a cure; or where Nature is quite able for the whole crisis as it proceeds through its course in certain definite stages, as in measles or benignant smallpox, &c.

"The second error is, DOING TOO MUCH, or the using of bloodletting and other active measures to such an extent as to damage the system more than the disease itself would have done."

Such, then, are the practical conclusions deduced by Hufeland from his long and varied experience, and which, after making allowance for the transcendentalism which obscures the meaning of two or three passages, seem to me to deserve the serious attention of your readers. Their general coincidence with the opinions lately advocated in your own pages is so striking, as to afford an additional presumption in favour of their truth; while, on the other hand, the objections urged with so little effect against you, seem to me equally invalid as applied to Hufeland. It would, for instance, be very difficult for any unprejudiced and reflecting person to read his summary with care, and still believe that his views lead to a *do-nothing* system of treatment. That Hufeland's principles are opposed to the *abuse* of active measures is indeed sufficiently manifest. But they are so far from condemning activity in a right direction and when really called for, that their proper application demands the constant exercise of greater watchfulness and discrimination in both observation and treatment, than are commonly met with. In like manner, it would be difficult for any candid and reflecting person to object to him, as has been done to me, that a treatment grounded on the indications of Nature must be erroneous, because if we never "thwart Nature," but, on the contrary, "always assist her efforts," we shall thereby be the means of hastening the death of the patient in those numerous cases in which "her efforts tend only to *destroy* life." This objection, indeed, is even more futile than the other, because it rests on a mere perversion of the proposition it is supposed to refute. It would be, not science, but downright folly in any one to contend seriously that we ought in any circumstances to *assist Nature in her destructive efforts;* and it is surprising that mere common sense should not have sufficed to prevent such a meaning being gravely ascribed to a few isolated sentences in your Review, even if their true meaning had not been rendered apparent by the context. Hufeland's statement, with which I concur, is, that in every disease there is an effort made by Nature *for the preservation of life and the restoration of health,* —an internal healing process,—without which recovery would be impossible, and which, as it takes place in accordance with fixed general laws, will be promoted or counteracted in proportion as the treatment resorted to shall, or shall not be in accordance with these laws, and with the *sanative* efforts of Nature. That such sanative efforts *are* made in every case is proved, not only by hourly experience, but by the recoveries occasionally effected by their unaided means, even in the deadliest epidemics of plague and cholera. Keeping in mind, then, that it is the sanative and not the destructive effort which is spoken of in the passages objected to, it still remains as consonant as ever to the soundest dictates of reason and experience, that the primary aim of the physician should be, by careful observation, to make himself acquainted with the plan and course of Nature, that he may be thereby enabled to co-operate with her and promote her efforts to effect a recovery.

As remarked in a former letter, the field of controversy would be greatly narrowed and the chances of agreement increased, if the objectors would consent to attach the same meaning to our propositions which we intend them to convey; and, doubtless, the discussions which have taken place, will have some effect in bringing about this most desirable end. But the differences between us are not altogether verbal or insignificant; they involve an important practical principle calculated to exert no small influence on the future progress of the healing art. But even if I were able, which I am not, to do full justice to the subject, it is not within the limits of a Review that it can be discussed with the requisite clearness and precision.

I remain, &c.,

Andrew Combe.

